# Synthesis and Performance Evaluation of Pulse Electrodeposited Ni-AlN Nanocomposite Coatings

**DOI:** 10.1155/2018/7187024

**Published:** 2018-01-24

**Authors:** Kamran Ali, Sivaprasad Narayana, R. A. Shakoor, Paul C. Okonkwo, Moinuddin M. Yusuf, Abdullah Alashraf, Ramazan Kahraman

**Affiliations:** ^1^Center for Advanced Materials (CAM), Qatar University, Doha, Qatar; ^2^Departments of Chemical Engineering, College of Engineering, Qatar University, Doha, Qatar

## Abstract

This research work presents the microscopic analysis of pulse electrodeposited Ni-AlN nanocomposite coatings using SEM and AFM techniques and their performance evaluation (mechanical and electrochemical) by employing nanoindentation and electrochemical methods. The Ni-AlN nanocomposite coatings were developed by pulse electrodeposition. The nickel matrix was reinforced with various amounts of AlN nanoparticles (3, 6, and 9 g/L) to develop Ni-AlN nanocomposite coatings. The effect of reinforcement concentration on structure, surface morphology, and mechanical and anticorrosion properties was studied. SEM and AFM analyses indicate that Ni-AlN nanocomposite coatings have dense, homogenous, and well-defined pyramid structure containing uniformly distributed AlN particles. A decent improvement in the corrosion protection performance is also observed by the addition of AlN particles to the nickel matrix. Corrosion current was reduced from 2.15 to 1.29 *μ*A cm^−2^ by increasing the AlN particles concentration from 3 to 9 g/L. It has been observed that the properties of Ni-AlN nanocomposite coating are sensitive to the concentration of AlN nanoparticles used as reinforcement.

## 1. Introduction

It is a compelling and intrinsic behavior of human to analyze and observe entities and objects of different subjects through visualization. In scientific community, it is considered valuable to get the visual effects of research output. It not only brings justification, authentication, and credibility to research but also makes it transparent to the audience. In this regard, scientists have always been thriving and struggling for advanced and sophisticated scanning methods and have successfully developed techniques such as SEM, TEM, AFM, XRD, Raman, 3D surface nanoprofilometry, XPS, and EDX [1–3]. Since the introduction of such techniques, the field of science has been greatly revolutionized. The surface morphology (roughness, uniformity, deformation, and defects), crystal structure, dimension analysis (thickness, size), compositional analysis (elemental, impurities, and stoichiometry), 3D geometrical features of coatings, nano/micromaterials, biological species, and so on have been analyzed with supreme transparency and accuracy with such sophisticated tools [4, 5]. Moreover, they have been effectively used for studying various phenomena and behaviors such as corrosion, creep, fatigue, crystal growth, and materials synthesis and have led the scientists towards great achievements [6–9].

Corrosion is considered as one of the major causes of failure of materials used in different applications particularly under harsh conditions. This detrimental mechanism is most often accompanied by wear phenomenon [10, 11]. The components used in production plants usually suffer from the gradual loss of materials which not only reduces the plant efficiency but also raises the maintenance cost. In most of the applications such as mining, mineral processing, and oil and gas industry, the impact of chemical attack is observed alongside the mechanical wear on the surface of the components. It leads us to the possible solutions and research opportunities in order to modify the surface properties by applying different types of coatings. This approach has been recognized as efficient and economical compared to the improvement and modifications of the entire material.

In recent years it has been focused not only on the need and requirement of solutions and strategies that can be implemented towards the control and inhibition of corrosion but also on imparting enhanced wear properties in steel components. This has been addressed and attempted using different strategies by renowned research groups across the globe [22–27]. One of the most recently sorted out approaches is the development of inorganic coatings over the surface of steel components [15, 28, 29]. These coatings have shown the core competence of simultaneously providing protection against high wear and corrosion. However, the true attributes and exact potential of such inorganic coatings have not been fully explored, and therefore, extensive and systematic research study is required to understand their complete attributes. Inorganic coatings with improved corrosion, abrasion, and wear resistance, produced by competitive and flexible routes and at competitive costs, are considered as strong candidates for providing solutions that can answer some of the most critical industrial requirements.

Among various inorganic coating options, nickel (Ni) based coatings have gained high attention due to their attractive properties. In this regard, Ni-P (nickel-phosphorus) and Ni-B (nickel-boron) coatings have been extensively studied for their promising attributes [11, 30–33]. Ni-B coatings present high hardness and excellent wear resistance but display inferior corrosion protection, whereas Ni-P coatings are well recognized because of superior anticorrosion properties but possess low hardness and inferior wear and erosion attributes. Inorganic coatings possessing simultaneously both attributes, high corrosion resistance and wear protection, have rarely been reported. This is a critical problem, which must be addressed, because the practical conditions in most industries involve aggressive species that induce corrosion which can be accelerated by mechanical factors, that is, stresses, wear, erosion, fatigue, and creep. The currently available coating solutions are not robust enough to solve this issue. Therefore, coatings with enhanced corrosion, wear, and erosion resistance produced through competitive, low cost, and flexible technologies are essential. To achieve these targets nanocomposite coatings are becoming a key part of the solution. Nanoparticles such as Al_2_O_3_, ZrO_2_, CeO_2_, Y_2_O_3_, TiO_2_, B_4_C, SiC, and carbon nanotubes have been widely proposed as additives to improve resistance against wear and corrosion [10, 23, 34–39].

Among the several processing methodologies which have been used for the synthesis of Ni based nanocomposite coatings, the pulse electrodeposition has turned out to be a promising coating deposition technique. Compared to one of the common trend of electroless deposition, the pulse electrodeposition method has major advantages such as ease of control of coating composition, low cost, high purity, high deposition rate, low processing temperature, stability of electrolyte at the operating temperature, uniform composition, microstructure, porosity, and grain size which result in coatings with superior properties. Moreover, a careful adjustment of optimized process parameters such as duty cycles, pulse current density, time ON/time OFF, and pulse frequency provides further freedom to accurately control the coating thickness and its composition [10, 40–42]. Due to the rapid developments in the field of coatings, implementation of new emerging advanced techniques is expected in the near future towards the synthesis of Ni based coatings [43–46].

In this study, aluminum nitride (AlN) has been selected as nanoadditive for Ni based coatings because of its great technological importance in a wide variety of applications. This semiconductor material has a wide-band gap and show attractive properties including thermal and chemical stability, biocompatibility, and good mechanical hardness [38]. Moreover, it has recently been recognized to possess exceptional intrinsic properties of wear and corrosion protection when added to various coatings. Previously, very few research studies have been conducted to understand and exploit the performance of Ni-AlN nanocomposite coatings. Therefore, it is of extreme importance to conduct a well systematic and detailed experimental study on Ni-AlN nanocomposite coatings being deposited through pulse electrodeposition. Unlike, the conventional electrodeposition, the technique of pulse electrodeposition involves more operational parameters such as frequency, duty cycle, and forward current. These additional parameters provide flexibility and freedom to control the deposition process with high accuracy. As a result, superior benefits are achieved such as the ability to control the coating's thickness and composition excellent surface morphology and high resistance to corrosion. To the best of our knowledge, pulse electrodeposition technique has not been employed before for the synthesis of Ni-AlN nanocomposite coatings. It is for the first time that pulse electrodeposited Ni-AlN nanocomposite coatings are studied in detail for their surface, mechanical, and electrochemical properties. It has been determined in this study that the Ni-AlN nanocomposite coatings produced through pulse electrodeposition process demonstrate simultaneous improvement in mechanical and anticorrosion properties making them suitable for many industries such as oil and gas, aerospace, seawater desalination, and automobile.

## 2. Experimental

### 2.1. Sample Preparation and Experiment Setup

**Figure 1:**
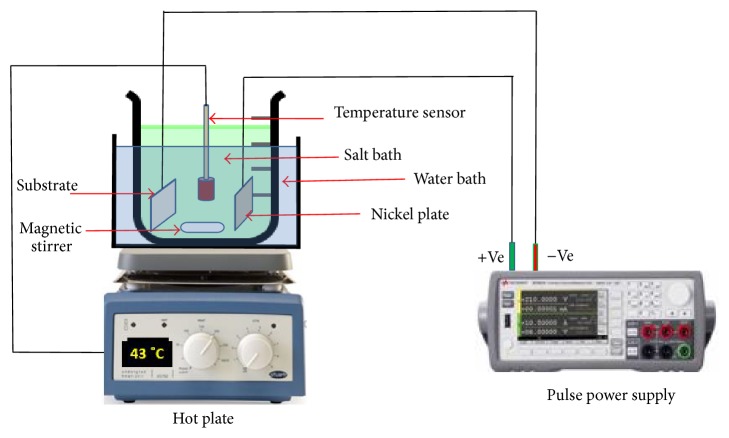
Experimental layout for Ni-AlN nanocomposite coatings developed by pulse electrodeposition process.

The Ni-AlN nanocomposite coatings were deposited on carbon steel substrates (Diameter 22 mm × 10 mm). The substrates were first mechanically cleaned using different SiC papers (220, 320, 500, 800, 1000, and 1200). The samples were then further processed through sonication for 30 minutes for degreasing using alkaline solution and acetone and were then carefully washed using distilled water. Finally, the substrate surfaces were activated using 20% HCl solution for 60 seconds and were then rinsed with distilled water. The edges and one face of the samples were insulated with epoxy tape in order to confine the electrodeposition of coatings to one selected surface of the sample. To proceed with the deposition, the carbon steel substrates were connected to the negative (cathode) and the nickel plate to the positive (anode) poles of the Dynatronix Pulse Power Supply. The schematic of the pulse electrodeposition experimental setup is presented in Figure 1.

The optimized bath chemical composition and coatings parameters for pulse electrodeposition bath are presented in Table 1. The substrate and the nickel sheet were placed parallel to each other in the plating bath. Various amounts of AlN powder, 3, 6, and 9 g/L, were added to the coating bath to investigate the effect of additive concentration on coating properties. The deposition was carried out at 43 ± 1°C for four cycles at 17 minutes/cycle. In order to avoid sedimentation and agglomeration of particles during the coating process, the nanocomposite coating bath was agitated at 300 rpm with a magnetic stirrer. Moreover, to ensure the uniform dispersion of the AlN particles, the coating bath was subjected to constant agitation for one hour before the start of the coating process using a magnetic stirrer.

### 2.2. Sample Characterization

The properties of the developed coatings were studied by conducting various characterization tests. The structural and phase analysis of the coatings were carried out through X-ray diffractometer (Rigaku, Miniflex2 Desktop, Tokyo, Japan) equipped with Cu K*α* radiations. Diffraction patterns were recorded at a scanning step of 0.02° in the 2*θ* range from 20° to 110°. The morphology of the coatings was studied using field emission scanning electron microscope (FE-SEM-Nova Nano-450, Netherland) and atomic force microscopy (AFM-USA). The FESEM images were taken using secondary electron (SE) mode. The compositional characterization was performed through Energy Dispersive X-Ray Spectroscopy. The mechanical properties (hardness, Young's modulus) were determined using MFP-3D Nanoindenter and Vickers microhardness tester (FM-ARS9000, USA). The measurement of microhardness was done using a 100 g load with a holding time of 10 seconds and then average of five measurements was taken as the resulting value. In corrosion tests, a three electrode flat electrochemical cell reported elsewhere was used [7]. Graphite was used as counter electrode, saturated calomel as reference electrode, and steel substrate as working electrode to study the electrochemical behavior of Ni-AlN nanocomposite coatings. Before the initiation of each corrosion test, deaeration was carried out using nitrogen gas for 2 h to flush out any contaminant gas that may have existed in the test cell. 3.5% NaCl was used as the corrosion medium and a saturation pH value of 7.2 was achieved. Corrosion tests were performed at ambient temperature. The potentiodynamic polarization experiments were carried out using a Gamry Reference Eco potentiostat at a scan rate of 10 mVmin^−1^ (0.167 mVs^−1^). Before the polarization tests, the working electrode was inserted in the electrochemical cell in contact with the test solution to attain a stable open circuit potential (OCP). After OCP stabilization the working electrode potential was polarized from a value of 250 mV below the open circuit potential to 250 mV above the open circuit potential. The Electrochemical Impedance Spectroscopy (EIS) experiments were conducted within a frequency range of 0.1 to 100 KHz, starting from the higher limit towards the lower one, and the rms signal was 10 mV.

## 3. Results and Discussion

### 3.1. Structural and Compositional Analysis

**Figure 2:**
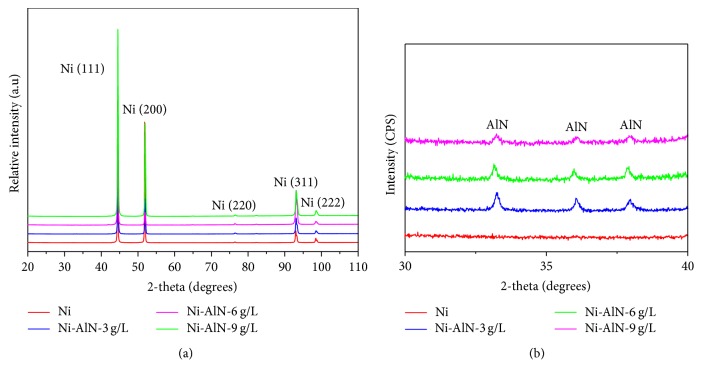
XRD analysis of electrodeposited nickel and Ni-AlN nanocomposite coatings: large scale XRD scan with high intensity nickel peaks (a) and small scale XRD scan showing AlN phases (b).

The synthesis of Ni-AlN nanocomposite coatings and the incorporation of AlN were confirmed through XRD and EDX analyses. The purity of the Ni matrix and the incorporation of AlN into the matrix are confirmed by corresponding peaks shown in Figures 2(a) and 2(b).

A comparison has been made to understand the relationship between AlN particle concentration and respective crystallography. The large scale scan shows diffraction peaks of (111), (200), (220), (311), and (222) for nickel at 45°, 52°, 77°, 93°, and 98°, respectively. Due to the high intensities of nickel peaks the signals for AlN are not apparent. However, the small scale XRD spectrum clearly reveals the presence of AlN nanoparticles (Figure 2(b)). Diffraction peaks were observed at 33°, 36°, and 38° corresponding to (100), (002), and (101) confirming the incorporation of AlN particles into the nickel matrix. Moreover, the continued presence of sharp high intensity nickel peaks indicates that addition of nanoparticles in low concentration does not influence much the crystalline structure of the nickel matrix. The observed results well satisfy the previously reported data by other research groups [38, 47, 48]. The XRD data were used to calculate the grain size (*L*) using the following Scherrer equation [49].

(1) $$\begin{array}{c} L=\frac{K\lambda }{\beta \left(2\theta \right)\times \cos\theta }. \end{array}$$

*L* is the mean size of the ordered (crystalline) domains and *K* is a dimensionless shape factor, with a value close to unity. The shape factor has a typical value of about 0.9 but varies with the actual shape of the crystallite; *λ* is the X-ray wavelength; *β* is the line broadening at half the maximum intensity (FWHM), after subtracting the instrumental line broadening, in radians. The calculated grain size for the developed coatings with different concentrations of AlN particles is presented in Table 2. The results clearly indicated a marked decrease with the increase in AlN particle concentration which is in accordance with the previous findings [32]. These findings indicate that the incorporation of AlN nanoparticles provides heterogeneous nucleation sites throughout the Ni matrix and blocks the grain growth [50, 51].

**Figure 3:**
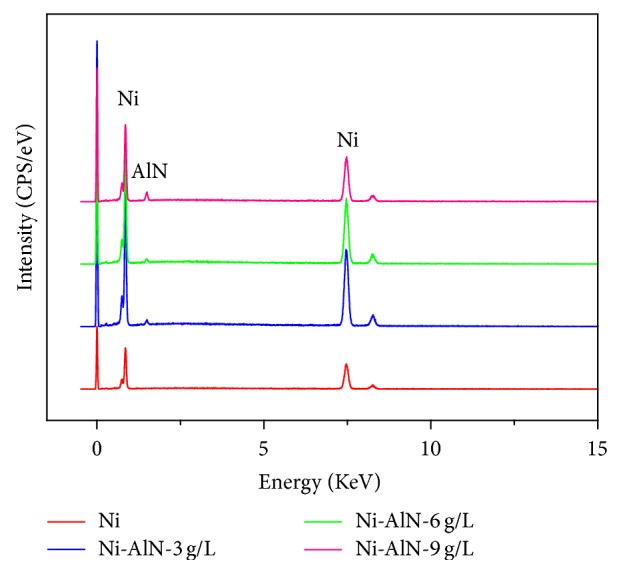
EDX spectrum of pulse electrodeposited Ni-AlN nanocomposite coatings.

The compositional analysis conducted through EDX also confirmed the incorporation of AlN nanoparticles in electrodeposited coatings as shown in Figure 3. It can be observed that an aluminum peak is detected in the spectrum which confirms the successful incorporation of AlN nanoparticles in the nickel matrix [38]. As evident from the presented data, the extent of AlN particles being induced in the coatings is prominently increased with the increase in AlN nanoparticle concentration used in the salt bath composition. The incorporation and subsequent deposition of AlN particles into the nickel matrix can be attributed to three possible mechanisms including mechanical interlocking, electrophoresis, and nanoparticle adsorption to cathode surface through Van der Waals attractive forces. During mechanical interlocking, the nanoparticles are being attached to the nickel ions which are then moved towards cathode due to electrophoresis. Once the particles arrive at the surface of the cathode, adsorption takes place and the nickel ions are reduced resulting in the encapsulation or deposition of nanoparticles into the nickel matrix [23, 35, 52].

### 3.2. Surface Morphology

**Figure 4:**
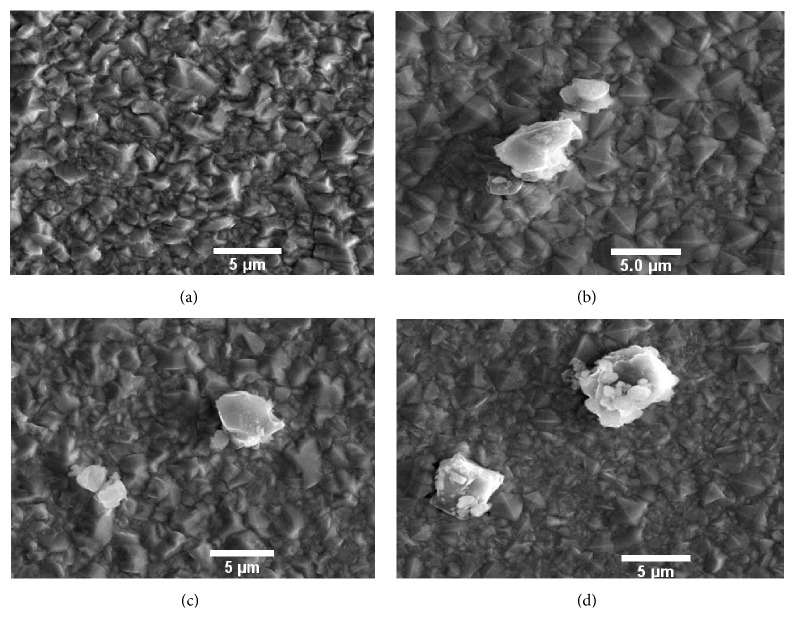
FESEM analysis of pulse electrodeposited nickel coating (a) and Ni-AlN nanocomposite coatings with AlN particle concentration of 3 g/L (b), 6 g/L (c), and 9 g/L (d).

The surface analysis of pulse electrodeposited Ni-AlN coatings has been carried out through field emission scanning electron microscope (FESEM) and atomic force microscopy (AFM). The FESEM images of pure nickel and Ni-AlN nanocomposite coatings for concentrations of 3, 6, and 9 g/L are presented in Figures 4(a), 4(b), 4(c), and 4(d), respectively.

The results show that pulse electrodeposition has produced intact coatings free of surface defects such as pores and cracks. Moreover, it reveals that homogenous and dense coatings were developed. Such morphological features are highly desirable as the corrosion phenomenon is highly reduced by restricting the permeation of water and other solvents towards the underlying metal substrate. The grain structure resembles the pyramid shape with pointed outgrowths which is believed to be beneficial for tribological applications because the tiny pockets among the pyramids can retain lubricant. Moreover, the pointed pyramid entities will result in small contact area which will enable the coatings to sustain wear phenomenon for longer period of times resulting in increase of coating's life cycle.

AlN particles agglomeration is also observed, which increases with the increase in concentration of AlN particles (Figures 4(b), 4(c), and 4(d)). Some of the agglomerates protrude above the nickel matrix which are believed to be helpful in the improvement of anticorrosive and mechanical properties of composite coatings [53]. The formation of agglomerates will improve the wear properties as AlN possess good intrinsic properties of mechanical hardness. The nickel matrix grain size was comparatively large for pure nickel coatings. However, a prominent and gradual decrease was observed in its size when the concentration of AlN particles was increased. It has been previously reported that mechanical properties are highly improved when the grain size refinement is achieved and thus believed to be a desirable feature of the coatings [32]. The refinement of the nickel matrix grain size can be attributed to the incorporation of AlN nanoparticles which facilitates the prevention of grain growth at the surface of cathode.

**Figure 5:**
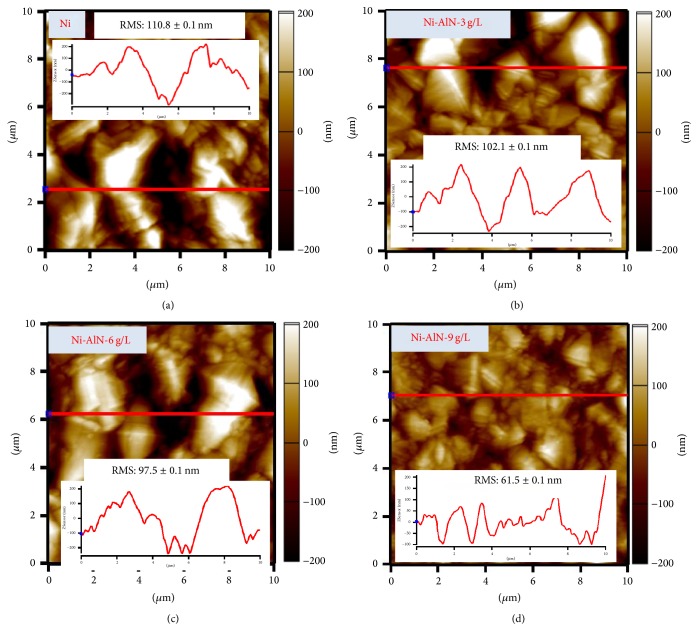
The 2D and X-profile of AFM analysis conducted for pure nickel and Ni-AlN nanocomposite coatings with increasing concentrations of AlN nanoparticles.

The morphological findings were further confirmed through AFM. The 2D and X-profile images of pure nickel and Ni-AlN nanocomposite coatings for increasing concentrations of AlN nanoparticles at 3, 6, and 9 g/L are shown in Figure 5.

The results well support the FESEM analysis confirming that the grain size has been reduced with the increasing concentration of AlN particles in the salt bath. The surface morphology is also studied quantitatively in terms of root-mean-square (RMS) roughness which is described as the average height deviation from the reference line. The RMS roughness for as deposited nickel coatings was found to be 110.8 ± 0.1 nm whereas continuously decreasing values of 102.1, 97.5, and 61.5 ± 0.1 nm were observed for Ni-AlN coatings with 3, 6, and 9 g/L concentrations of AlN particles, respectively. The decrease in surface roughness of Ni-AlN nanocomposite coatings with increasing amount of AlN nanoparticles is essentially because of gradual decrease in grain size which helps the particles to be uniformly packed together to reduce the surface roughness [54]. Furthermore, the AFM analysis confirmed that the developed coatings were free of defects such as cracks and pores.

**Figure 6:**
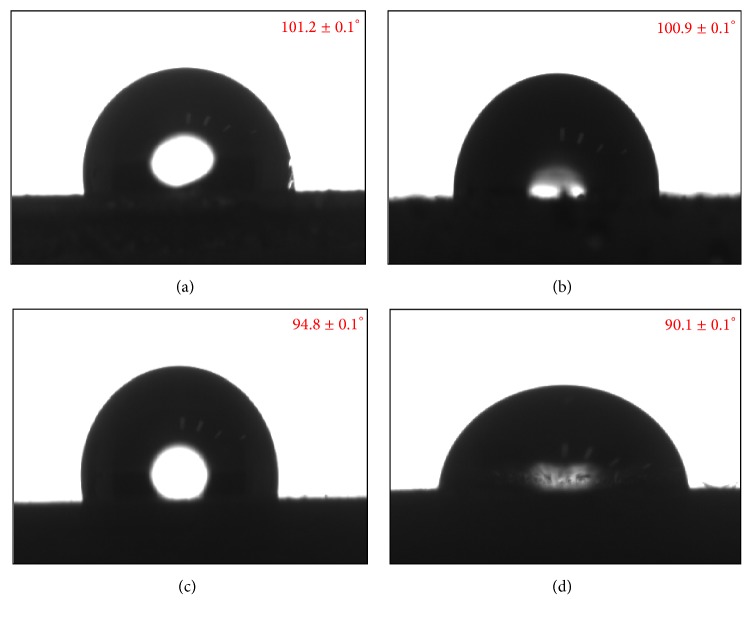
The contact angle of pure nickel coating (a) and Ni-AlN nanocomposite coatings with increasing concentrations of AlN nanoparticles with 3, 6, and 9 g/L, showing contact angle of 100.9 ± 0.1° (b), 94.8 ± 0.1° (c), and 90.1 ± 0.1° (d), respectively.

One of the highly desirable features of coatings towards the application of corrosion inhibition and protection is the hydrophobicity. As the fundamental factor and source of corrosion are water, therefore, a key strategy is to develop systems or functional coatings which are hydrophobic or most preferably super hydrophobic in nature so that there is none or minimum contact of water/moisture with the metal surface. In this regard, the contact angle analysis has been conducted for the currently developed Ni-AlN nanocomposite coatings and the results are shown in Figure 6. It can be observed that the pure nickel coatings have the highest tendency to repel water with water contact angle of 101.23°. However, the contact angle was decreased when the AlN nanoparticles were incorporated in the coatings. As evident from the morphological analysis including FESEM and AFM, the surface roughness of the coatings is decreased with the increase in AlN particles concentration. The surface structure and morphology have a strong influence on hydrophobicity effect. Previous reports have confirmed that rough surfaces demonstrate higher levels of hydrophobicity which is also observed in the current research study as the contact angle is reduced with increasing concentration of AlN nanoparticles and consequent reduction of coating's surface roughness [55]. The surface roughness of pure nickel coatings is comparatively higher than the Ni-AlN nanocomposite coatings and thus demonstrated superior hydrophobic properties.

### 3.3. Mechanical Properties

**Figure 7:**
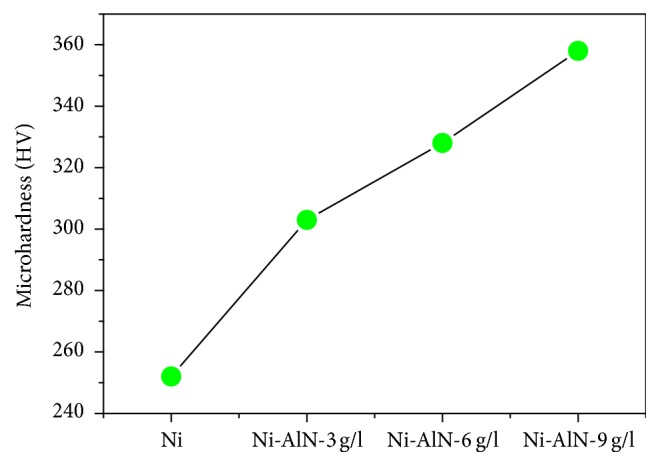
Hardness of the pulse electrodeposited coatings versus pure nickel and increased concentration of AlN nanoparticles.

The hardness of pulse electrodeposited Ni-AlN nanocomposite coatings was analyzed to determine the influence of AlN nanoparticles incorporation on the mechanical behavior of Ni-AlN nanocomposite coatings. The study was conducted through Vickers microhardness tester using a 100 g load with a holding time of 10 seconds and the results are presented in Figure 7. Each value is average of five readings. It can be observed from the data that hardness of the coatings has been greatly improved with the increasing concentration of AlN nanoparticles in the pulse electrodeposited coatings. The nickel coatings demonstrated hardness of HV 252.134, whereas increased hardness of HV 303.448, HV 328.134, and HV 357.768 was observed for the coatings with AlN concentrations of 3, 6, and 9 g/L, respectively. The hardness of some of the previously reported nickel based composite coatings was summarized in Table 3 for comparison.

**Figure 8:**
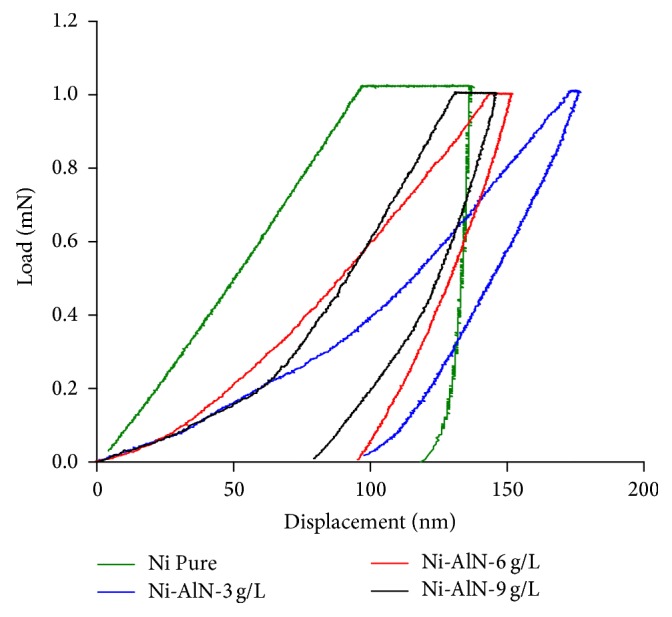
Nanoindentation profiles of pure nickel and Ni-AlN nanocomposite coatings with increasing concentrations of AlN nanoparticles.

The influence of AlN concentrations on the mechanical behavior of Ni-AlN composite coatings was further analyzed through nanoindentation. The loading and unloading profiles of nanoindentation for the comparative analysis of as prepared nickel and Ni-AlN coatings are presented in Figure 8.

It can be observed from the nanoindentation study that the AlN nanoparticle has a marked effect on the hardness of the composite coatings. The two main parameters are area under the curve and the depth of penetration. The pure nickel coatings showed larger area under the curve and also the highest indentation depth of ~120 nm. It clearly indicates that pure nickel coatings possess less resistance to indentation. Therefore, such coatings are not suitable for the applications involving high wear. In order to address this problem and to enhance the mechanical attributes of the coatings, hard AlN particles are incorporated into the nickel matrix. The addition of AlN particles in the coatings with a concentration of 9 g/L resulted in a major improvement in hardness and showed superior resistance to indentation having depths of ~78 nm. The improvement of hardness properties of the developed Ni-AlN composite coatings by the addition of nanoparticles is mainly attributed to the mechanism of grain refinement and dispersion hardening effects. The incorporated nanoparticles act as obstacles towards the movement of dislocations due to which the hardness is improved [10, 35].

The observed behavior satisfies the previous findings from microhardness tests confirming that hardness of the nanocomposite coatings is well enhanced with the increase in concentration of AlN nanoparticles. Similar trends have been reported by different research groups supporting the idea of nanoparticle incorporation for the improvement of mechanical properties [56, 57]. The nanoindentation loading and unloading profiles were also used to conduct quantitative analysis for calculation of mechanical hardness of nanocomposite coatings. The pure nickel coatings showed a hardness value of 2.21 GPa and a continuous increase to 2.50, 2.66, and 3.14 GPa was observed for composite coatings with increasing concentrations of 3, 6, and 9 g/L, respectively.

It can also be observed that the indentation profiles of the deposited coatings possess kinks except that of the pure nickel. These kinks originate due to the presence of pores or multiphase structure of the coatings. In case of pores, there might occur a microcollapse underneath the indentor which may cause abrupt variation in the stress-strain analysis. In the same way when the indentor scans a second phase particle, then the slope in load-displacement profile will fluctuate [58]. As observed from FESEM analysis the coatings were free of defects such as pores so these kinks can be attributed to the AlN second phase particles in the matrix. The current results also follow the mentioned mechanism as the pure nickel coatings showed smooth and free of kinks profile. However, due to the presence of second phase AlN particles, several kinks can be observed in the nanoindentation profiles of nanocomposite coatings.

### 3.4. Corrosion Behavior

**Figure 9:**
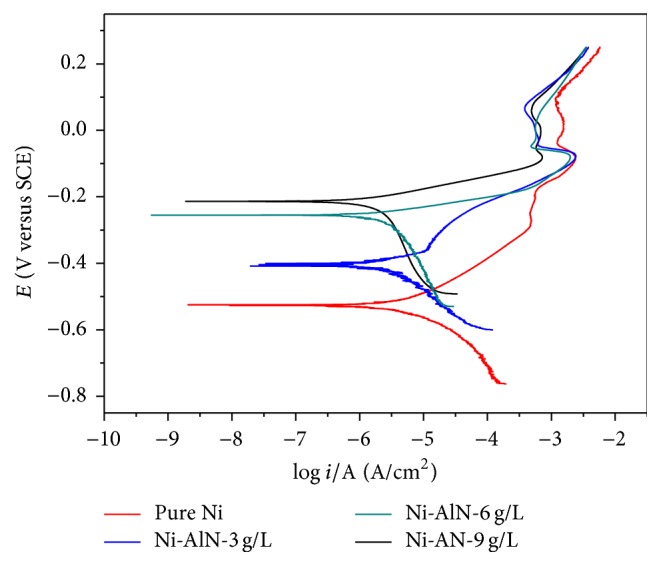
Potentiodynamic polarization of pure nickel and Ni-AlN nanocomposite coatings with 3, 6, and 9 g/L AlN particles using 3.5 wt.% NaCl aqueous solution at a scan rate of 0.167 mV s^−1^. The polarization curve for pure nickel coating is included to establish a detailed comparison. *i*_corr_, *β*_*a*_, *β*_*c*_, and *E*_corr_ are obtained from the potentiodynamic polarization curves and are summarized in Table 4.

Figure 9 illustrates the potentiodynamic polarization (*E* versus log⁡*i*) curves obtained in aqueous 3.5 wt.% NaCl solution for pure nickel and Ni-AlN coatings for increasing concentrations of 3, 6, and 9 g/L of AlN particles.

According to Table 4, *i*_corr_ of the nickel coating was approximately 2.57 *μ*A cm^−2^. Enhancement of the coating performance by addition of AlN resulted in decreasing corrosion current density as the percentage of the AlN increases. There are parameters that could be responsible in the corrosion resistance of coating. Occurrence of pores that are usually considered as active sites for commencement of pitting corrosion and discontinuity in the coating layer due to deposited phase boundaries are considered as important factors in the corrosion resistance. As previous results indicated [59] the incorporation of second phase particles resulted in improved coatings avoiding defects such as pores and therefore contributing towards decrease in current density (high corrosion resistance) with increasing concentration of AlN particles as observed in Table 4. In addition, the incorporation of AlN nanoparticles may have also gradually blocked the pores and reduced the active area of nickel matrix with their increasing amount which has resulted in improved anticorrosion properties [10].

**Figure 10:**
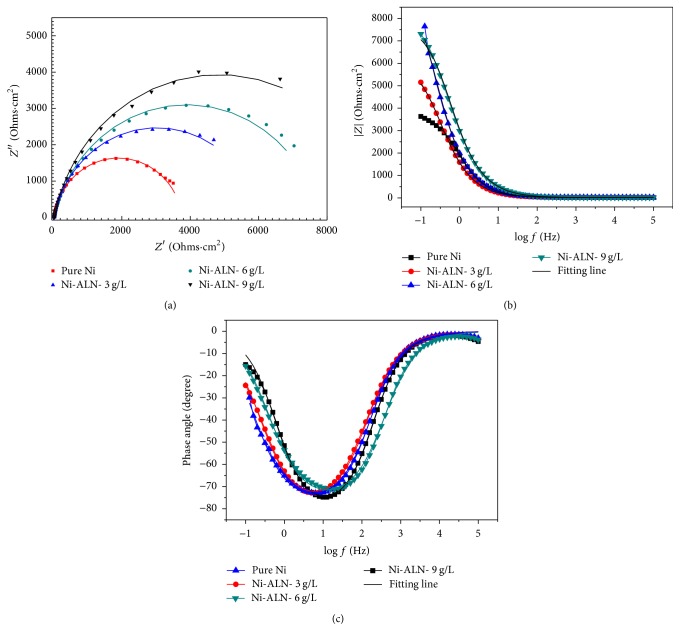
Measured (dotted lines) and fitted (solid lines) EIS data represented in Nyquist (a), bode (b), and phase angles plots (c) format for Ni, Ni-3, Ni-6, and Ni-9 g/L AlN coatings in aqueous 3.5 wt.% NaCl solution within a frequency range of 0.1 to 100 kHz.

The EIS characterization of the prepared pure nickel and Ni-AlN nanocomposite coatings with 3, 6, and 9 g/L AlN particles was carried out in 3.5 wt% NaCl aqueous solution to study their corrosion behavior. EIS Nyquist, bode, and phase angles plots of Ni, Ni-3, Ni-6, and Ni-9 g/L AlN coatings and the corresponding fitting lines (solid lines) are shown in Figure 10. The semicircles of the Nyquist graphs of the concentration of AlN nanoparticles are larger in size comparing with the pure Ni semicircle. There is a depressed capacitive loop seen at high frequency in Nyquist plot. This means that the corrosion of Ni-AlN coating in the explored environment is controlled by charge transfer mechanism. The deviation of the capacitive loop from a complete semicircle might be due to the heterogeneity and microroughness of the working electrode. It can be noted that the phase angle at high frequencies delivers a general knowledge of the implementation of Ni-AlN coating. Increasing the concentration of Ni-AlN coating shifts the phase angle at high frequencies to a more negative value.

**Figure 11:**
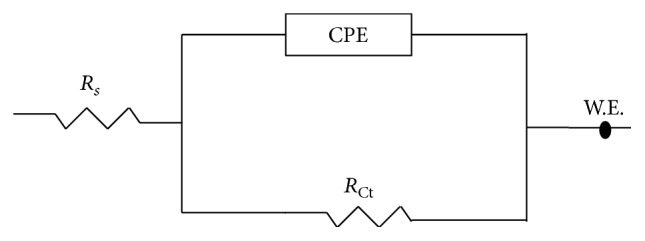
The electrical equivalent circuit used for fitting EIS data.

The equivalent circuits used for fitting the measured EIS data are shown in Figure 11. In the analysis, simple Randles electrical equivalent circuit with one time constant was used in which the ideal capacitor was replaced by a constant phase element. In the diagram, *R*_*s*_ represents the solution resistance; CPE in the circuit is the constant phase element; *R*_ct_ stands for the charge transfer resistance.

The EIS parameters derived from fitting the measured impedance spectra using the equivalent circuit shown in Figure 11 are listed in Table 5.

All the values were obtained using the Gamry Echem Analysis Software technique [60]. The increase in *R*_ct_ indicates that the Ni-AlN nanocomposite coatings are more protective as compared to pure Ni coatings. Moreover, this protectiveness increases with increasing amount of AlN used as reinforcement. These results show a good agreement with Tafel analysis parameters shown in Table 4. It has been previously reported that the presence of surface defects (microcracks, porosity, etc.) in the Ni-W-P matrix influences the corrosion resistance of the coating [61]. Liu et al. showed that the SiC nanoparticles when embedded in the Ni-W-P matrix filled in crevices, gaps, and microholes result in smaller pores which makes the passive film more compacted [6]. Therefore, addition of AlN to Ni matrix (3 to 9 g/L) increases the corrosion resistance of Ni coatings. As a comparison, the Ni-AlN-9 g/L nanocomposite coatings showed better corrosion resistance compared to Ni-AlN-6 g/L, Ni-AlN-3 g/L, and pure nickel coating.

## 4. Conclusions

High quality Ni-AlN nanocomposite coatings have been developed through pulse electrodeposition process. A significant influence of addition of AlN nanoparticles is noticed on the surface, mechanical, and anticorrosion properties of Ni coatings. SEM and AFM analyses confirm that addition of AlN nanoparticles into Ni matrix results in the formation of homogenous, dense, and pyramid microstructure. It is further noticed that the surface roughness of Ni-AlN nanocomposite coatings decreases with the increasing amount of AlN nanoparticles. The hardness of Ni-AlN nanocomposite coatings increases with increasing amount of AlN nanoparticles. Moreover, Ni-AlN nanocomposite coatings demonstrate superior anticorrosion properties when compared to Ni coatings. Finally, the hardness and corrosion protection ability of Ni-AlN nanocomposite coatings increase with the increase in concentration of AlN particles. A simultaneous improvement in mechanical and anticorrosion properties of Ni coatings is achieved by the addition of AlN nanoparticles to form Ni-AlN nanocomposite coatings suggesting their potential applications in many industries.

## Figures and Tables

**Table 1:** Optimized bath composition and plating parameters for pulse electrodeposited Ni-AlN nanocomposite coatings.

S/N	Bath constituents	Composition in 1 L	Composition in 100 ml
(1)	Nickel sulphate hexahydrate	240 g	24 g
(2)	Nickel chloride hexahydrate	45 g	4.5 g
(3)	Boric acid	30 g	3 g
(4)	AlN powder	3, 6, and 9 g	0.3, 0.6, and 0.9 g
*Operating conditions*			
(1)	pH	4 ± 0.1	
(2)	Temperature	43 ± 1°C	
(3)	Deposition time	68 min (4 cycles each consists of 17 minutes)	
(4)	Current density	50 mA/cm^2^	
(5)	Bath agitation	300 rpm	
(6)	Frequency	800 Hz	
(7)	Forward current	0.45 Amps	
(8)	Duty cycle	50%	
(9)	*T* (TON + TOFF) ms	1.25	
(10)	TON ms	0.63	
(11)	TOFF ms	0.63	

**Table 2:** Calculated crystalline domain size for the pulsed electrodeposited Ni-AlN coatings.

Identity	2*θ*	*β*	*L* (A°)
Ni	44.4537	0.0936	0.3999
Ni-AlN-3 g/L	44.5337	0.1248	0.2995
Ni-AlN-6 g/L	44.4618	0.1404	0.2666
Ni-AlN-9 g/L	44.5180	0.1560	0.2396

**Table 3:** Hardness of the previously reported nickel based composite coatings.

Constituents	Content of particles	Hardness	Method	Application	Reference
Ni-WC	10.5 ± 0.4 vol%	378 ± 18 HV	Cold spray	Wear protection	Alidokht et al. [12]
Ni-TiO_2_	1 g/L	9.98–12.06 GPa	Electrodeposition	Corrosion	Birlik et al. [13]
Ni-TiN	10–30 g/L	3.23 GPa	Electrodeposition	Corrosion	Parhizkar et al. [14]
Ni-CeO_2_	30 g/L	436 HV	Electrodeposition	Corrosion	Zeng et al. [15]
Ni-Si_3_N_4_	12 g/L	720 HV	Electrodeposition	Corrosion	Kasturibai and Kalaignan [16]
Ni-SiO_2_	5–45 g/L	800–850 HV	Electrodeposition	Wear protection	Li et al. [17]
Ni-ZrO_2_	3.37 wt.%	462 HV	Electrodeposition PRC plating	Wear protection	Wang et al. [18]
Ni-Al_2_O_3_	30 g/L	426 HV	Electrodeposition	Wear protection	Jeyaraj et al. [19]
Ni-SiC	0.8–0.15 wt.%	247–270 HV	Thermal spray	Wear protection	Lanzutti et al. [20]
Ni-graphene	0.1–0.4 g/L	~207–224 HV	Electrodeposition	Wear protection	Chen et al. [21]

**Table 4:** Potentiodynamic polarization parameters for pure nickel and Ni-AlN coatings with 3, 6, and 9 g/L AlN particles using 3.5 wt.% NaCl aqueous solution at the scan rate of 0.167 mV s^−1^.

Sample	*E*_corr_, V	*β*_*a*_, mV decade^−1^	*β*_*c*_, mV decade^−1^	*i*_corr_ (*μ*A cm^−2^)
Ni	−0.526	0.089	0.098	2.57
Ni-AlN-3 g/L	−0.405	0.105	0.091	2.15
Ni-AlN-6 g/L	−0.255	0.279	0.045	1.95
Ni-AlN-9 g/L	−0.214	0.343	0.036	1.29

**Table 5:** EIS parameters obtained for corrosion of Ni, Ni-3, Ni-6, and Ni-9 g/L of AlN coating after being tested in 3.5 wt% NaCl aqueous solution at open circuit potential within a frequency range of 0.1 to 100 kHz at 25°C.

Sample	*R*_ct1_, KΩ·cm^2^	*R*_*s*_, Ω·cm^2^	CPE2, (Ss^*n*^cm^−2^)	*n* (0 < *N* < 1)
Ni	3.753	18.54	118.3 × 10^−6^	0.858
Ni-AlN-3 g/L	6.012	21.93	95.2 × 10^−6^	0.913
Ni-AlN-6 g/L	7.76	22.02	79.6 × 10^−6^	0.875
Ni-AlN-9 g/L	9.621	23.87	56.72 × 10^−6^	0.872
